# Reared Microgastrine Wasps (Hymenoptera: Braconidae) from Yanayacu Biological Station and Environs (Napo Province, Ecuador): Diversity and Host Specialization

**DOI:** 10.1673/031.009.3101

**Published:** 2009-06-02

**Authors:** James B. Whitfield, Josephine J. Rodriguez, Paul K. Masonick

**Affiliations:** Entomology Department, University of Illinois, Urbana, IL 61801

**Keywords:** parasitoids, Lepidoptera, host ranges

## Abstract

The microgastrine braconid wasps recovered up through 2007 by the NSF-sponsored rearing project “Caterpillars and Parasitoids of the Eastern Andes in Ecuador” are summarized in terms of their host specialization and faunistic uniqueness. Two hundred fifty eight rearings of caterpillars resulted in records of Microgastrinae, distributed among 14 genera (*Apanteles* Förster, *Choeras* Mason, *Cotesia* Cameron, *Diolcogaster* Ashmead, *Distatrix* Mason, *Dolichogenidea* Viereck, *Exix* Mason, *Glyptapanteles* Ashmead, *Hypomicrogaster* Ashmead, *Papanteles* Mason, *Parapanteles* Ashmead, *Protapanteles* Ashmead, *Sathon* Mason and *Venanus* Mason). Eleven records of hyperparasitoids of Microgastrinae are also summarized; *Mesochorus* Gravenhorst (Ichneumonidae) and Perilampidae are both recorded. The results are compared to those recovered by surveys in other parts of the world, especially by the Janzen-Hallwachs survey of the Area de Conservación Guanacaste (ACG) in Costa Rica. An annotated list of microgastrine genera not yet recorded at Yanayacu, but which we expect to eventually find there based on extrapolation from their known geographic distributions, is provided.

## Introduction

Microgastrine braconid wasps are abundant, diverse endoparasitoids of caterpillars throughout essentially all terrestrial habitats on the globe. As a consequence, they have figured strongly in countless ecological and agricultural studies including over 100 pest biological control projects. *Cotesia* species, for example, have been used in ecological studies of the consequences of metapopulation structure (van Nouhuys and Lei 2004; van Nouhuys 2005), tritrophic interactions (Harvey et al. 2005), the formation of cryptic species (Kankare et al. 2005) and the relationships between mating systems and sex determination mechanisms (de Boer et al. 2007a, b, 2008; Zhou et al. 2006).

Because Microgastrinae disrupt the biology of their host caterpillars in highly specific ways, they have also been used as tools for studying insect nutritional physiology, endocrinology and immunology (summarized in Beckage 1993, 2002; Beckage and Gelman 2004). Mediating these host interactions is a mutualistic relationship with endosymbiotic polydnaviruses, which help to overcome the host's immune system. These viruses have recently generated great interest in terms of functional genomics (Espagne et al. 2004; Kroemer and Webb 2004; Webb and Strand 2004) and evolution (Whitfield 1997b, 2000, 2002, 2003; Whitfield and Asgari 2003; Turnbull and Webb 2002; Espagne et al. 2004; Desjardins et al. 2005; Webb et al. 2006; Stoltz and Whitfield 2009). A strong systematic foundation for this growing wealth of comparative data is highly desirable. Indeed, the wasp/virus biology community already makes heavy predictive use of the limited phylogenetic information available for microgastrines and their relatives (Lapointe et al. 2007, Bezier et al. 2009).

Building a phylogenetic foundation for Microgastrinae has been elusive for several reasons. First, the high species richness of the subfamily (nearly 2,000 described species), especially in tropical regions, has meant that despite significant taxonomic effort on the group, thousands of species remain to be described. Because of their relatively small body size (1.5–4.0mm), microgastrines have, for the most part, been ignored by general entomologists, so that accumulation of new data has tended to center around highly specialized scientific projects. The group's classification was largely developed by a small number of individuals who dedicated many years to their study. The late W. R. M. Mason was the first to develop a phylogeny-based classification for Microgastrinae (Mason 1981) and related subfamilies (Mason 1983; Whitfield and Mason 1994). Although his work has been criticized (Austin 1990; Maetô 1996; Walker et al. 2000; Achterberg 2002), Mason's classifications remain widely used today.

As systematists and field biologists have continued to apply Mason's classification to the wealth of new taxa being discovered, significant problems have arisen, and it has become evident that our understanding of microgastrine phylogeny needs improvement. Several large genera (e. g. *Diolocogaster, Glyptapanteles*) are either polyphyletic, or at least paraphyletic as currently delineated (Choi and Whitfield in review), and Mason's tribal groupings conflict with more recent phylogenetic results (Walker et al. 1990; Mardulyn and Whitfield 1999; Whitfield et al. 2002; Banks and Whitfield 2006). Several genera have recently been described (Whitfield 1995; Choi and Whitfield 2006), in part to classify species that do not fit well into Mason's (1981) classification. Large scale phylogenetic studies of Microgastrinae based on multiple genes (Banks and Whitfield 2006; Murphy et al. 2008) are currently underway in several laboratories. These promise to set the stage for a more robust classification in the near future.

The neotropical Microgastrinae fauna has, until recently, been especially poorly known, both taxonomically and biologically (Whitfield 1997a). Several small to moderatesized genera have recently been reviewed or revised: *Alphomelon* (Deans et al. 2003); *Austrocotesia* (Valerio and Whitfield 2005); *Deutenxys* (Whitfield and Oltra Moscardo 2004); *Diolcogaster* and related genera (Choi and Whitfield 2006; Choi in review); *Distatrix* (Grinter et al. this issue); *Exoryza* (Valerio et al. 2004); *Hypomicrogaster* (A. A. Valerio, personal communication); *Parapanteles* (Valerio et al. 2005b; Valerio and Whitfield 2009); *Prasmodon* (Valerio et al. 2005a); and *Teremys* (Valerio and Whitfield 2003).

New species have been described in several of the larger genera - e. g., *Apanteks* (Whitfield et al. 2001); *Glyptapanteles* (Whitfield et al. 2002a); *Microplitis* (Janzen et al. 2003), and *Sendaphne* (Scatolini and Penteado-Dias 1999) - without full revisions having been attempted. The vast bulk of neotropical microgastrines remain undescribed.

In contrast to the temperate fauna, however, knowledge of the host biology/ecology of neotropical microgastrines is accumulating faster than they can be taxonomically studied, due to systematic rearing surveys of Lepidoptera larvae at several sites. Principal among these surveys are the long-term studies of Janzen and Hallwachs in the Area de Conservación Guanacaste (ACG) in northwest Costa Rica (http://janzen.sas.upenn.edu/caterpillars/database.lasso), the similarly designed survey of Dyer and Gentry at the La Selva Biological Station in Costa Rica (http://www.tulane.edu/%7eldyer/lsacatold/Families.htm), and the more recently initiated study in the eastern Andes of Ecuador led by Dyer, Gentry, Greeney and Walla (http://www.tulane.edu/%7eldyer/lsacat/ecuador/index.htm). For literally hundreds of microgastrine wasp taxa, we now have detailed information about their host caterpillars, caterpillar host plants, and habitat preferences, even before the wasp species have been formally named. Our group has begun an attempt to alleviate this problem by organizing the reared material to make it available for comprehensive systematic study, and by undertaking taxonomic research on particular genera.

In this paper we briefly describe faunistic findings concerning Microgastrinae from the last of these surveys, based at the Yanayacu Biological Station in Napo Province, Ecuador (http://www.yanayacu.org/). The generic composition recovered from Yanayacu, and host preference patterns within the genera, are compared to those known from Costa Rica and elsewhere in the world. For each genus, we provide a habitus photograph and we illustrate diagnostic characters so that the wasps can be readily recognized. We provide an annotated list of genera that might be reasonably be expected to occur with continued sampling in the eastern Andes. We also summarize the hyperparasitoids reared from Microgastrinae in the survey. Formal descriptions of taxa and detailed accounts of species diversity remain to be completed in the future, after the samples have been DNA barcoded and subjected to intense taxonomic study. It is already evident from the ACG microgastrines that accurate rearing data and DNA barcodes are revealing many cryptic, host-specific species that might not have been suspected from mass-collected material (Smith et al. 2008).

### Genera of Ecuadorian Microgastrinae recovered to date

After more than 50,000 individual caterpillar rearings, 258 have yielded microgastrine wasps. These are distributed among 14 genera. We summarize findings in each genus below.

### *Apanteles* Förster (Figure 1)

With an estimated 2,000 species, *Apanteles* Förster, the largest microgastrine genus (Mason 1981), is typically one of the dominant genera reared in caterpillar inventories. Of the 61 reared records of *Apanteles* from Ecuador, 29 are from pyraloid hosts. The remaining hosts (with number of records in parentheses) are from Geometridae (11), Noctuidae (3), Hesperiidae (2), Arctiidae (2), Choreutidae (1), Saturniidae (1) and yet to be determined families (12). This is consistent with what is known for the North American fauna, where the majority of *Apanteles* attack many concealed host caterpillar in Pyraloidea, Tortricoidea, Gelechioidea and Tineoidea especially, although exceptions have been reared from Macrolepidoptera (Mason 1981). *Apanteles* from the ACG survey in Costa Rica have also been reared from a diversity ofsmall concealed hosts. A large percentage of hosts there, however, are hesperiid caterpillars (http://janzen.sas.upenn.edu/caterpillars/database.lasso), which seem to be less common and less diverse at Yanayacu.

**Figure 1.**
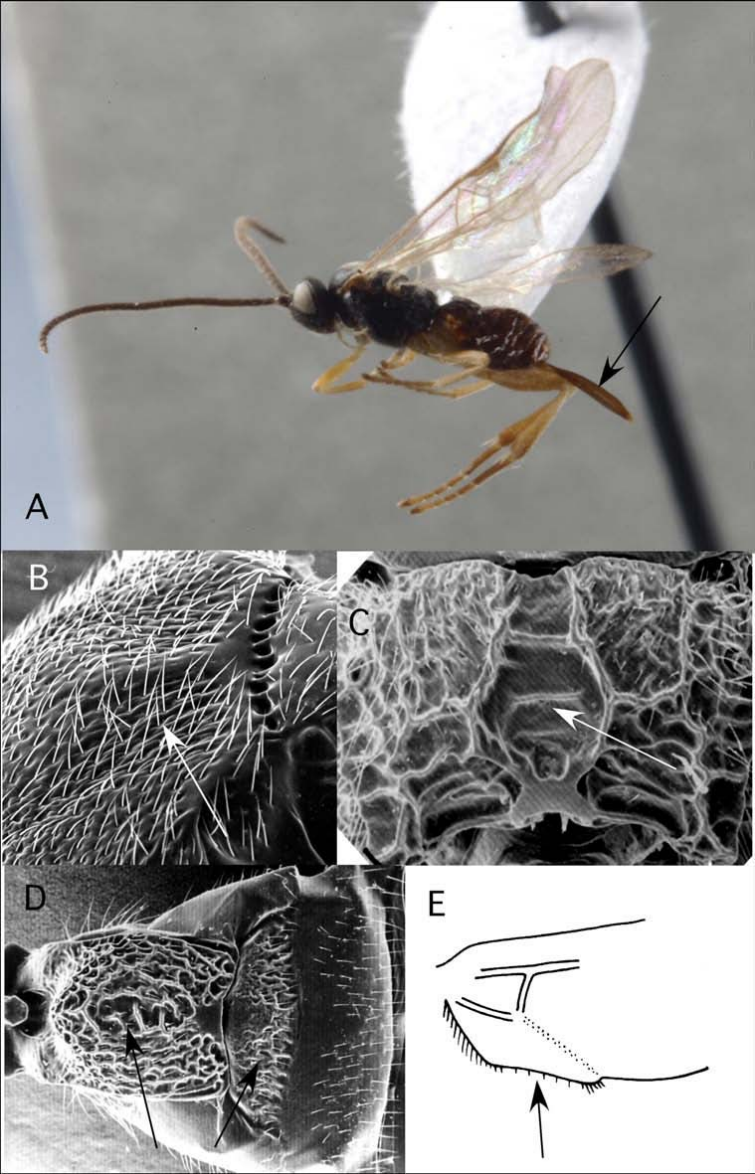
*Apanteles* Förster. A: lateral habitus of a female from Yanayacu, showing long ovipositor and sheaths. B-E: diagnostic features of the genus. B: posterior of mesonotum, showing longitudinally confluent punctation. C: propodeum, showing medial areola. D: anterior metasomal tergites, showing medial depression on tergite I and strongly transverse tergite II. E: vannai lobe at base of hindwing, showing flattened and sparsely fringed distal margin (after Whitfield 1997a).

*Apanteles* can be recognized among the New World microgastrines by the following combination of features (see also Figure 1): forewing with second r-m vein absent, so that the small areolet (second submarginal cell) is open distally (as in Figure 3D); hindwing with vannal lobe distally flattened and with reduced fringe of hairs (Figure 1E); punctation of posterior part of mesonotum breaking down into more confluent longitudinal sculpturing, especially submedially (Figure 1B); propodeum with oval, pentagonal, hexagonal or anteriorlyopen medial areola (Figure 1C); first metasomal tergite usually with medial subapical depression (Figure 1D) and second metasomal tergite strongly transverse, often with convex or sinuate posterior margin (Figure 1D); ovipositor and sheaths long and exserted (Figure 1A), manipulatable via a medially desclerotized hypopygium (subgenital plate). The genus is easily confused with the related but less diverse *Dolichogenidea*, which differs in having distinct punctures posteriorly on the mesonotum, and a convex and evenly fringed hindwing vannai lobe (Figure 6E).

### Choeras Mason (Figure 2)

To date, this genus has been reared only once in the Ecuador survey, from a pyralid feeding on *Piper* (Piperaceae). In general, the genus seems to specialize on Pyraloidea (Mason 1981), but the single host recorded in the ACG survey is in the Thyrididae. *Choeras* is seldom common, except in more temperate regions of South America (e. g. Argentina).

**Figure 2.**
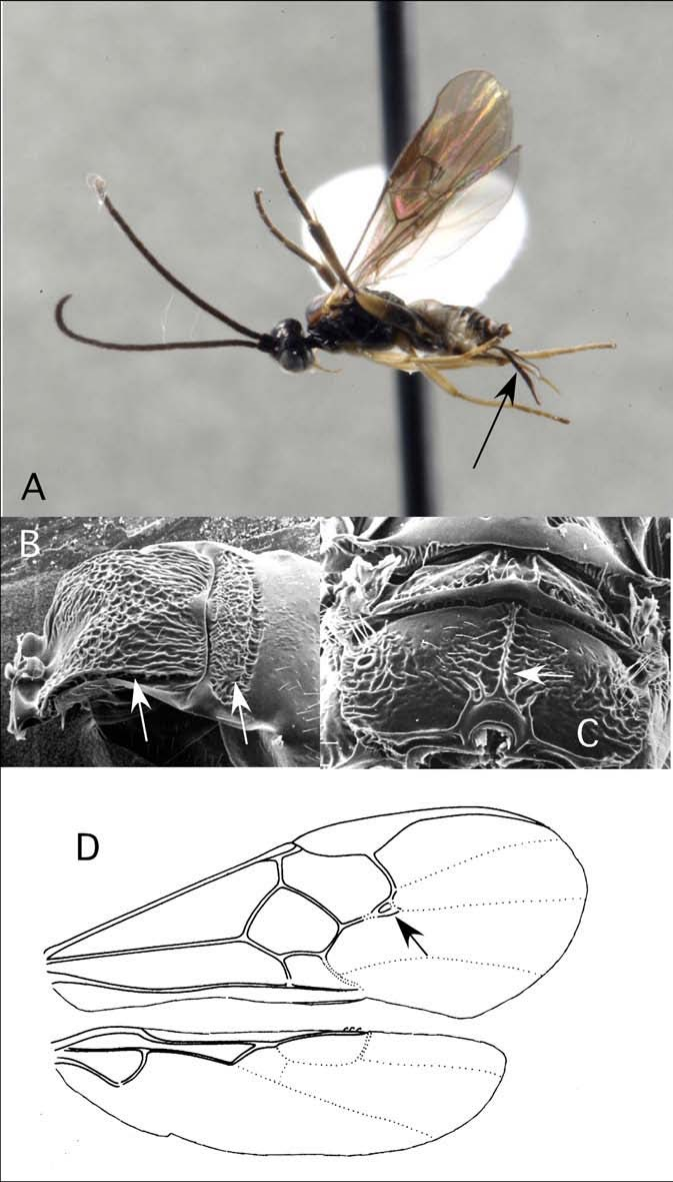
*Choeras* Mason. A: lateral habitus of a female from Yanayacu, showing long ovipositor and sheaths. B-D: diagnostic features of the genus. B: anterior metasomal tergites, showing straight lateral margins of tergite I and strongly transverse tergite II. C: propodeum, showing medial carina. D: wing venation, showing distally closed small areolet in forewing (after Whitfield 1997a)

*Choeras* is recognizable by the following combination of features (see also Figure 2): forewing with second r-m vein typically present, so that a small, often inconspicuous, areolet is evident (Figure 2D); propodeum with a medial longitudinal carina rather than an areola (Figure 2C); first metasomal tergite typically with rather straight lateral margins, and second tergite strongly transverse (Figure 2B); ovipositor and sheaths long and exserted (Figure 2A), manipulatable via a medially desclerotized hypopygium.

### *Cotesia* Cameron (Figure 3)

This is one of the largest microgastrine genera, with hundreds of species, although it tends to be a more dominant faunal component in temperate regions worldwide. *Cotesia* species attack exposed larvae from many families of larger moths and butterflies. In the Ecuador survey, they have been recovered 20 times, from Noctuidae, Geometridae and Saturniidae. These are typical hosts elsewhere in the World as well.

**Figure 3.**
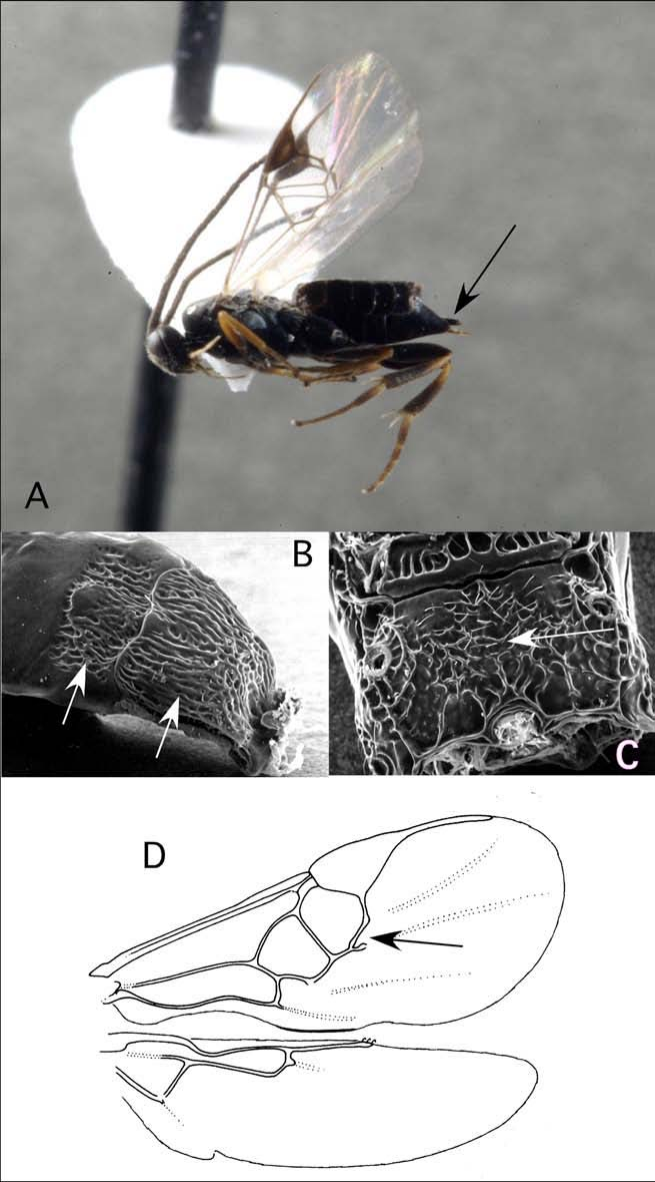
*Cotesia* Cameron. A: lateral habitus of a female from Yanayacu, showing short ovipositor and sheaths. B-E: diagnostic features of the genus. B: propodeum, showing medial carina and course background sculpturing. C: anterior metasomal tergites, showing course sculpturing and quadrate form. E: wing venation, showing distally open areolet in forewing (after Whitfield 1997a).

*Cotesia* is recognizable among microgastrines by the following combinations of features (see also Figure 3): forewing with second r-m vein absent, so that the small areolet is open distally (Figure 3D); propodeum coarsely sculptured with medial carina rather than medial areola (Figure 3C); first and second metasomal tergites usually rather quadrate in form and coarsely sculptured (Figure 3B); ovipositor and sheaths short and barely exserted (Figure 3A). The genus is easily confused with the distantly related *Parapanteles*, which differs in having a medial areola (Figure 11B) that is sometimes superimposed on similar coarse background sculpturing.

### Diolcogoster Ashmead (Figure 4)

This genus has only been reared once in the survey, from a pyralid larva. The genus is quite diverse, both in terms of species richness and biology/morphology, especially in lowland tropics worldwide. It is possible that the Yanayacu Station is at too high an elevation (2000+m) for many of the Amazonian *Diologaster* species. Nevertheless, we expect additional species to be recovered as sampling continues, from a wide variety of hosts, extending beyond Pyraloidea into Noctuoidea, Geometroidea and Papilionoidea. The ACG survey has recovered dozens of *Diolcogaster* species.

**Figure 4.**
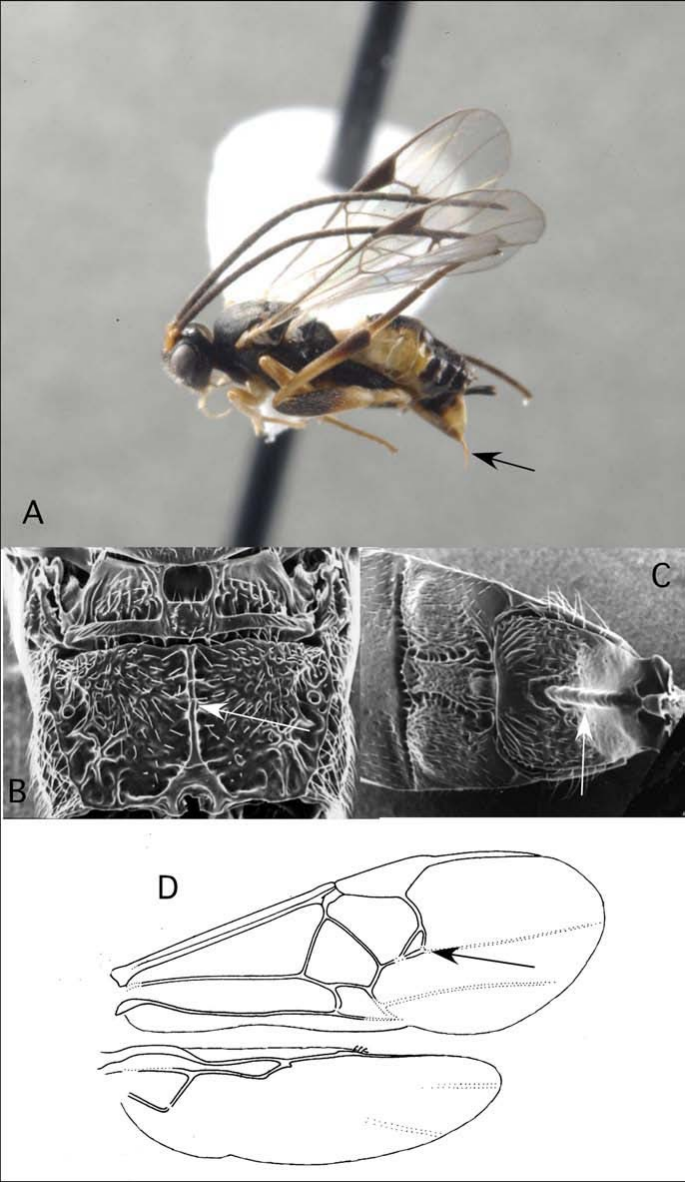
*Diolcogoster* Ashmead. A: lateral habitus of a female from Yanayacu, showing short ovipositor and sheaths. B-D: diagnostic features of the genus. B: propodeum, showing medial areola. C: anterior metasomal tergites, showing medial groove on tergite I. D: wing venation, showing distally closed small areolet in forewing (after Whitfield 1997a).

*Diolcogaster*, highly variable in form and color pattern, is generally recognizable by the following combination of features (see also Figure 4): forewing with second r-m vein present, so that a small areolet is formed (Figure 4D); propodeum with medial carina rather than medial areola (Figure 4B); hind coxa relatively large, extending to or beyond end of third metasomal tergite; first metasomal tergite anteriorly with medial groove (Figure 4C); ovipositor and sheaths short and barely exserted (Figure 4A). The genus is probably not monophyletic, without synonymizing several other small genera within it (Choi and Whitfield in review).

### *Distatrlx* Mason (Figure 5)

*Distatrix* is geographically widespread, but usually uncommon. The genus is typically reared from Geometridae. At least two species occur at Yanayacu. The first is a parasitoid of geometrids, especially *Eois*, which feeds on *Piper* spp. (Piperaceae). The second is a gregarious parasitoid of the rare nymphalid butterfly *Antirrhea adoptiva porphyrosticta* Watkins, which feeds on the cloud forest bamboo *Chusquea scandens* Kunth (Poaceae) (see Grinter et al. this issue and Greeney et al. this issue). We have two confirmed records of the first species, and one of the second, but expect more as the hosts are intensively surveyed. The ACG survey has recovered several additional species from geometrids and noctuids (see Grinter et al. this issue), consistent with earlier host records from the Nearctic Region (Whitfield and Scaccia 1996; Whitfield et al. 1999).

**Figure 5.**
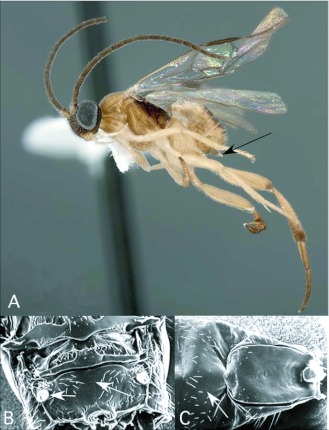
*Distatrix* Mason. A: lateral habitus of a female from Yanayacu, showing short ovipositor and sheaths (after Grinter et al. this issue). B-C: diagnostic features of the genus. B: propodeum, showing medially smooth, unsculptured surface and enlarged spiracles. C: anterior metasomal tergites, showing weakly margined tergite II.

*Distatrix* can generally be recognized by the following combination of features (see also Figure 5 as well as Grinter et al. this issue): forewing with second r-m vein absent, so that the small areolet (second submarginal cell) is open distally (as in Figure 3D); propodeum weakly sculptured, sometimes with enlarged spiracles (Figure 5B); first tergite and second metasomal tergites usually rather smooth, the second subtriangular with poorly defined margins posteriorly (Figure 5C); ovipositor and (nearly bare or minutely setose) sheaths short and barely exserted (Figure 5A). The genus could be confused with *Glyptapanteles*, which differs in that the first metasomal tergite is more strongly narrowed posteriorly, the margins of the second metasomal tergite are better defined, and the ovipositor sheaths exhibit normal setae apically.

### *Dolichogenidea* Viereck (Figure 6)

There are eight records of this genus from Ecuador -- six from pyraloids and two from unknown host families. This is consistent with what is known about *Dolichogenidea* in other regions, where they typically attack pyraloids, tortricoids, tineoids, gelechioids and other small concealed hosts (Mason 1981). In the ACG survey, this genus has been recorded from Pyralidae and Crambidae, although the majority of hosts are from the Thyrididae, Mimallonidae, and Elachistidae (http://janzen.sas.upenn.edu/caterpillars/database.lasso). Despite their large size, members of these moth families typically feed in concealment.

**Figure 6.**
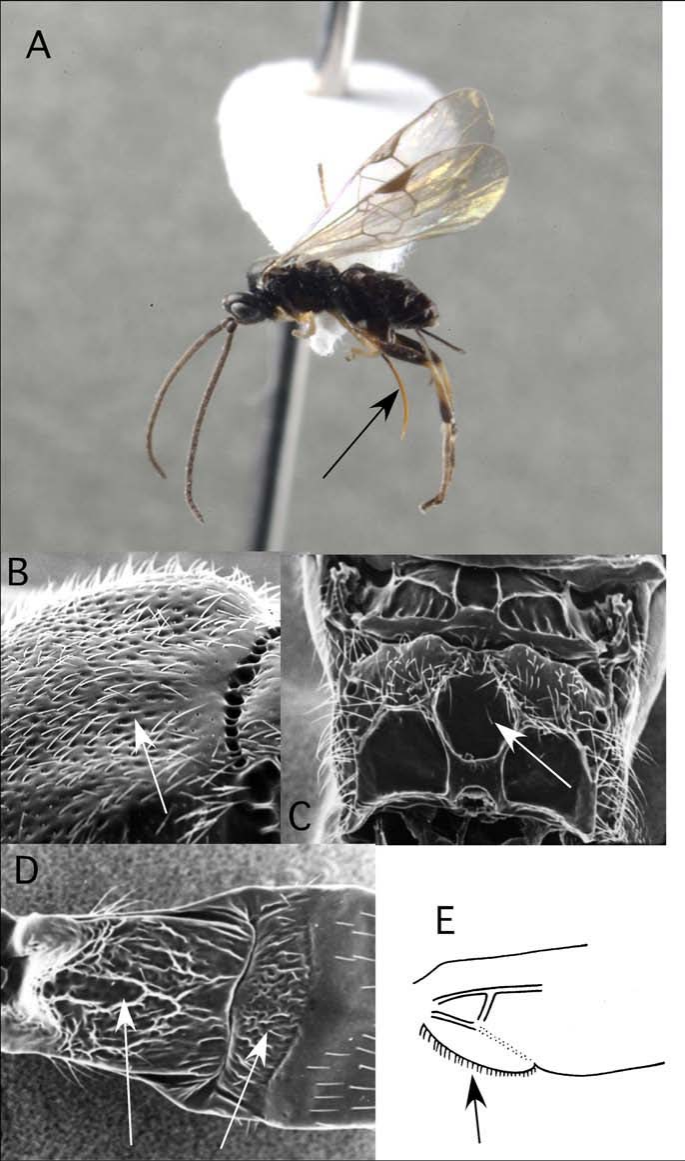
*Dolichogenidea* Viereck. A: lateral habitus of a female from Yanayacu, showing long ovipositor and sheaths. B-E: diagnostic features of the genus. B: posterior of mesonotum, showing distinct punctation. C: propodeum, showing medial areola. D: anterior metasomal tergites, showing medial depression on tergite I and strongly transverse tergite II. E: vannai lobe at base of hindwing, showing convex and evenly fringed distal margin (after Whitfield 1997a).

*Dolichogenidea* can be recognized among the New World microgastrines by the following combination of features (see also Figure 6): forewing with second r-m vein absent, so that the small areolet is open distally (as in Figure 3D); hindwing with vannai lobe distally convex, bearing an even fringe of hairs (Figure 6E); punctation of posterior part of mesonotum remaining distinct submedially (Figure 6B); propodeum with oval, pentagonal, hexagonal or occasionally poorly defined medial areola (Figure 6C); first metasomal tergite usually broad, with a medial subapical depression (Figure 6D) and second metasomal tergite strongly transverse, often with a convex or sinuate posterior margin (Figure 6D); ovipositor and sheaths long and exserted (Figure 1A), manipulatable via a medially desclerotized hypopygium. The genus is easily confused with the closely related but more diverse *Apanteles*, which differs in having longitudinal sculpturing posteriorly on the mesonotum (Figure 1B), and a flattened and sparsely fringed hindwing vannal lobe (Figure 1E).

### *Exix* Mason (Figure 7)

This genus has been reared once by the survey, from a geometrid feeding on *Palicourea* sp. (Rubiaceae). The only other rearing record for this small New World genus (containing six described species, including two from Ecuador) is from *Syngrapha* sp. (Noctuidae) in British Columbia (Mason 1981). The ACG survey has not recovered *Exix*.

**Figure 7.**
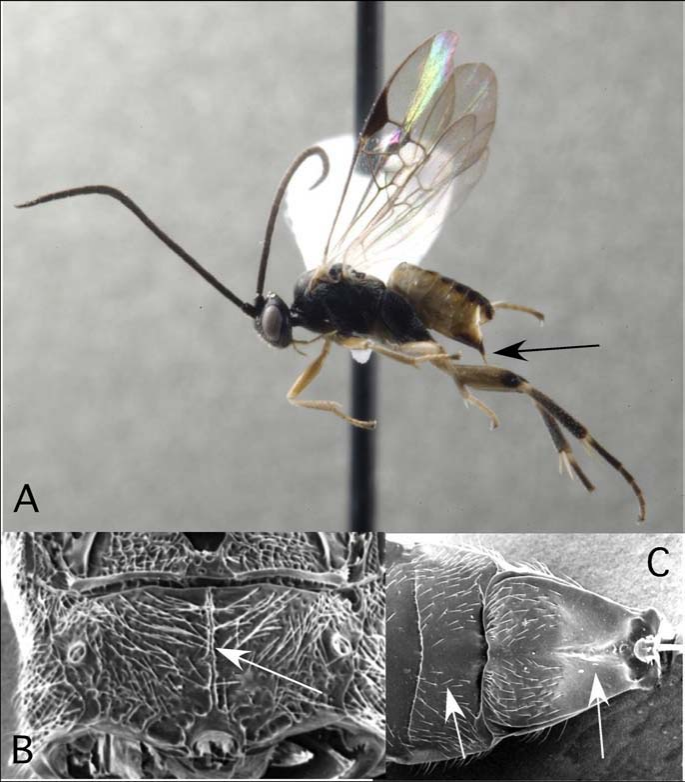
*Exix* Mason. A: lateral habitus of a female from Yanayacu, showing short ovipositor and sheaths. B-C: diagnostic features of the genus. B: propodeum, showing medial carina. C: anterior metasomal tergites, showing anterior medial groove on tergite I and weakly sculptured roughly rectangular tergite II.

*Exix* can be distingushed by the following combination of features (see also Figure 7): forewing with second r-m vein present, so that a small areolet is formed (as in Figure 4D); propodeum finely sculptured with medial carina rather than medial areola (Figure 7B); first and second metasomal tergites usually rather quadrate in form and either finely sculptured or polished; the first tergite with a longitudinal groove antero-medially (Figure 7C); ovipositor and sheaths short and barely exserted (Figure 7A).

The genus should possibly be synonymized under *Diolcogaster*, with which it shares a number of features.

### *Glyptapanteles* Ashmead (Figure 8)

This is one of the most diverse microgastrine genera in the neotropics, especially in lowland regions. We have 94 records so far from Ecuador, reared from a variety of Apatelodidae, Arctiidae, Geometridae, Limacodidae, Noctuidae, Nymphalidae, Pieridae, Pyralidae and Saturniidae. The limacodid records are unusual for *Glyptapanteles*, but otherwise the Ecuadorian host preferences that are typical for the genus elsewhere.

**Figure 8.**
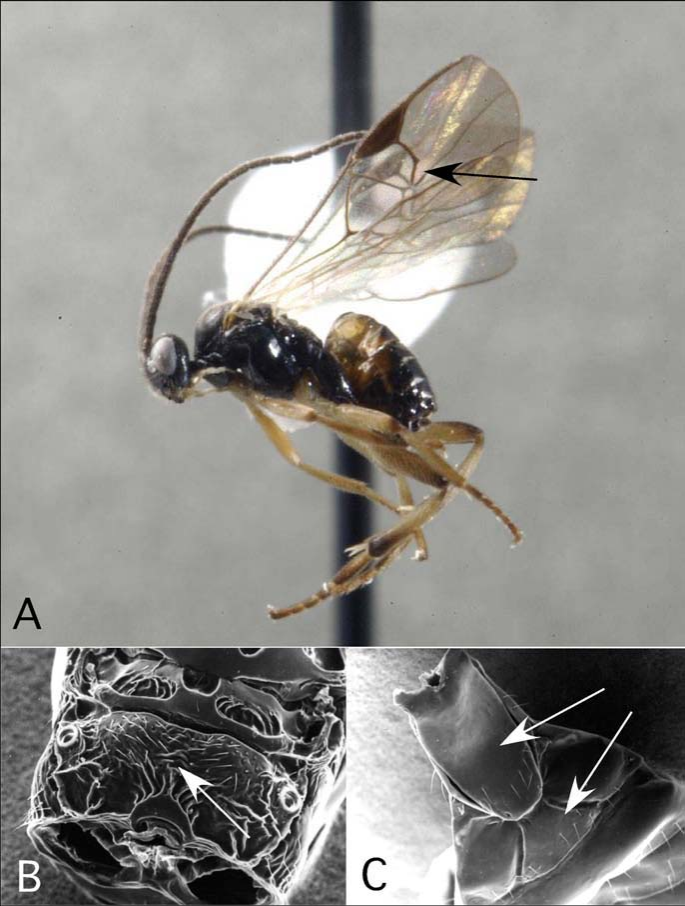
*Glyptapanteles* Ashmead. A: lateral habitus of a female from Yanayacu, showing distally open areolet in forewing. B-C: diagnostic features of the genus. B: propodeum, showing hint of medial carina and absence of an areola. C: anterior metasomal tergites, showing posteriorly narrowing first tergite and posteriorly broadening second tergite.

*Glyptapanteles* can be distinguished from other microgastrine genera by the following combination of features (see also Figure 8): forewing with second r-m vein absent, so that the small areolet is open distally (as in Figure 3D); propodeum coarsely sculptured with medial carina rather than medial areola, or nothing medially (Figure 8B); first metasomal tergite narrowing posteriorly, second metasomal tergite broadening posteriorly and often nearly triangular (Figure 8C); ovipositor and sheaths short and barely exserted. The genus is distinctive, although it could be confused with some *Distatrix*.

### *Hypomicrogaster* Ashmead (Figure 9)

*Hypomicrogaster*, another genus with high species richness in the neotropics, also occurs in the Holarctic Region (A. A. Valerio personal communication). In temperate zones, the species are usually recorded as solitary parasitoids almost exclusively emerging from small concealed-feeding caterpillars, including leafminers. It is becoming evident that the host spectrum of *Hypomicrogaster* is much broader in the neotropics, where it attacks many lepidopteran groups. Neotropical taxa also differ in that they often develop gregariously. Several dozen species of *Hypomicrogaster* have been recorded from the ACG survey. The Ecuador project has recorded the genus seven times, from a variety of Choreutidae, Lasiocampidae, Noctuidae and Pyralidae.

**Figure 9.**
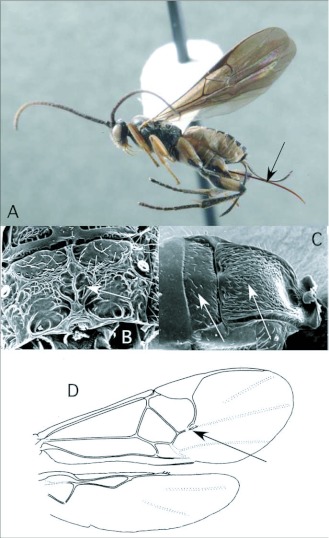
*Hypomicrogaster* Ashmead. A: lateral habitus of a female from Yanayacu, showing long ovipositor and sheaths. B-D: diagnostic features of the genus. B: propodeum, showing oval medial areola bisected by a medial carina. C: anterior metasomal tergites, showing broad tergite I and relatively polished and transverse tergite II. D: wing venation, showing distally closed small areolet in forewing (after Whitfield 1997a).

*Hypomicrogaster* is relatively variable morphologically, but can be distinguished by the following combination of features (see also Figure 9): forewing with second r-m vein typically present, so that an especially small, often inconspicuous, areolet is evident (Figure 9D); propodeum with a medial longitudinal carina, but usually also with a superimposed areola (Figure 9B); first metasomal tergite typically broad, and second tergite less sculptured and strongly transverse (Figure 9C); ovipositor and sheaths usually long and exserted (Figure 2A), manipulatable via a medially desclerotized hypopygium; a few species possess shorter ovipositors, but otherwise fit the above description.

### *Papanteles* Mason (Figure 10)

This is a small genus, restricted to the Neotropics (Mason 1981). The two rearings of this genus from Ecuador are from quite different hosts - a choreutid feeding on a fern, and a pyralid feeding on *Begonia* (Begoniaceae). The few rearings from ACG are from Crambidae (Pyraloidea).

**Figure 10.**
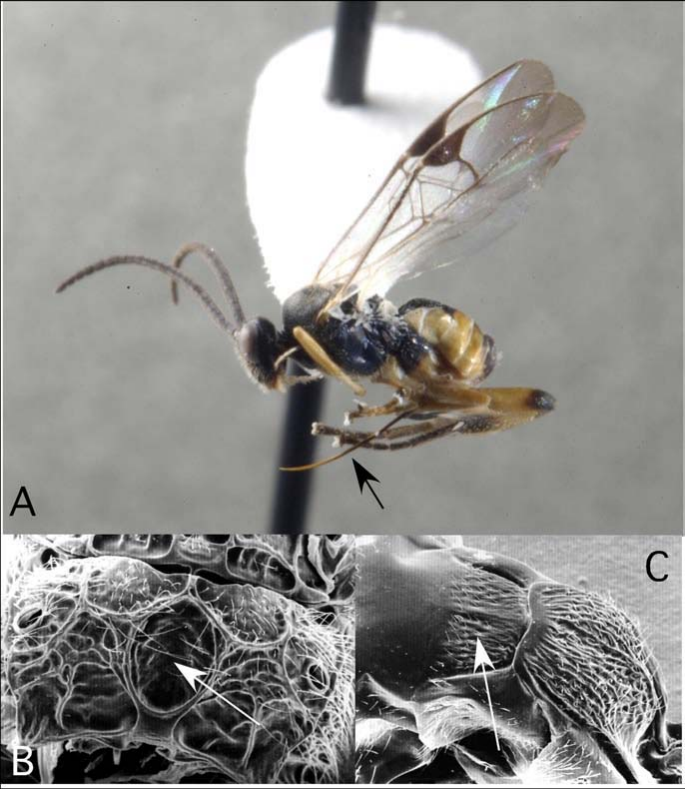
*Papantdes* Mason. A: lateral habitus of a female from Yanayacu, showing long ovipositor and sheaths. B-C: diagnostic features of the genus. B: propodeum, showing well-formed medial areola. C: anterior metasomal tergites, resembling many male *Apanteles* in form.

*Papanteles* resembles *Apanteles*, but is distinguishable from it, and from microgastrines generally, by the following combination of features (see also Figure 10): forewing with second r-m vein present, so that a small areolet is evident (as in Figure 9D); propodeum with well-defined oval medial areola (Figure 10B); first and second tergites resembling *Apanteles* but with tergite II less transverse (Figure 10C); ovipositor and sheaths long and exserted (Figure 10A), manipulatable via a medially desclerotized hypopygium. The closed forewing areolet immediately separates this genus from *Apanteles* and *Dolichogenidea*.

### *Parapanteles* Ashmead (Figure 11)

Until recently, this mostly New World genus was considered to be small and relatively rare, since it does not appear frequently in Malaise trap samples. In the past, the genus was often confused with *Cotesia*. In the ACG rearing project, at least two dozen *Parapanteles* species have now been reared, from a broad taxonomic spectrum of macrolepidopteran hosts. In the Ecuador project, the genus has now been reared 43 times, from a wide variety of hosts, including Apatelodidae, Arctiidae, Geometridae, Noctuidae, Notodontidae, Nymphalidae, Pyralidae and Saturniidae. A revision of *Parapanteles* has been recently completed (Valerio and Whitfield 2009), but that treatment must be considered provisional in light of the rapid (and continuing) increase in our knowledge of the genus.

**Figure 11.**
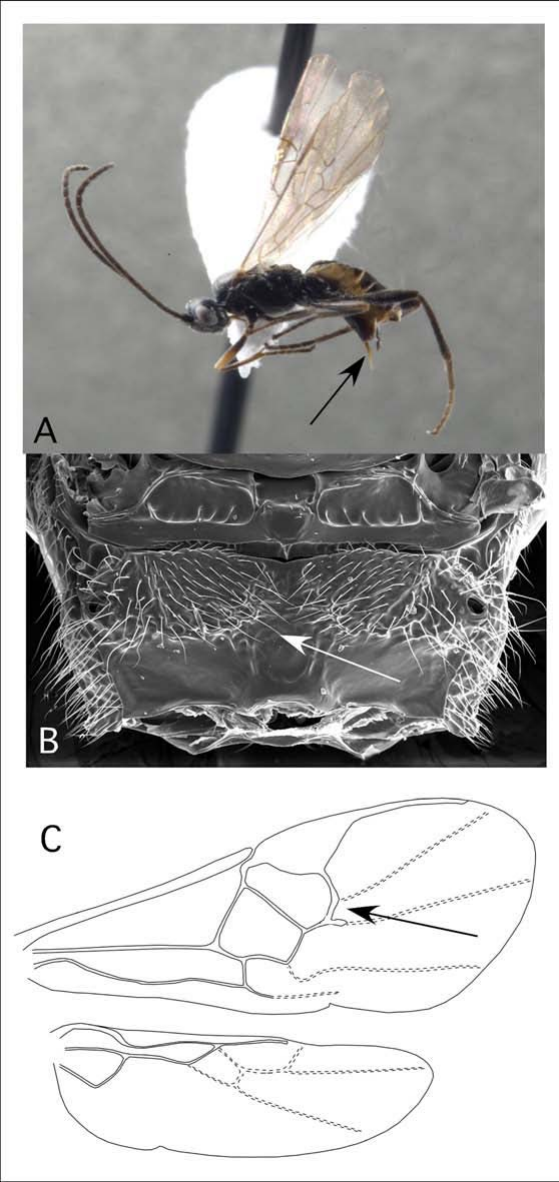
*Parapanteles* Ashmead. A: lateral habitus of a female from Yanayacu, showing short ovipositor and sheaths. B-D: diagnostic features of the genus. B: propodeum, showing weakly indicated pentagonal medial areola. C: wing venation, showing distally open small areolet in forewing. B & C after Valerio et al. (2005).

*Parapanteles* has always been a puzzling genus, and though it has frequently been confused with *Cotesia*, the two are only distantly related (Whitfield et al. 2002). It has the following combination of features (see also Figure 11): forewing with second r-m vein absent, so that the small areolet is open distally (Figure 11 C); propodeum often rather coarsely sculptured (as in *Cotesia*) but with illformed medial areola rather than medial carina (Figure 11B); metasomal tergites I and II usually rather quadrate in form and coarsely sculptured (as in *Cotesia*, but sometimes more closely resembling *Dolichogenidea*); ovipositor and sheaths short and barely exserted (Figure 3A).

### *Protapanteles* Ashmead (Figure 12)

This small genus is most common in the Holarctic region, and its appearance (once) among the Ecuador samples was a surprise. At first we doubted the generic determination, but can now confirm the record. The identity of its host is uncertain. The caterpillar, which fed on *Solarium* sp. (Solanaceae), did not develop into an adult. *Protapanteles* is usually reared as a solitary parasitoid from Geometridae (Mason 1981).

**Figure 12.**
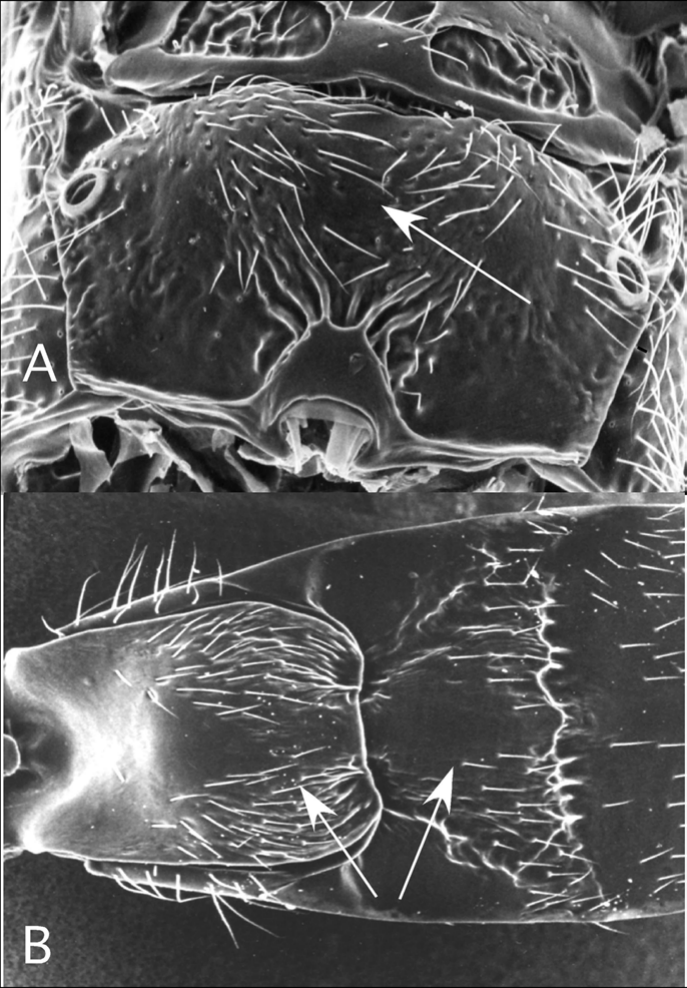
*Protapanteles* Ashmead, diagnostic features of the genus. A: propodeum, showing relatively weak sculpturing as in *Glyptapanteles*. B: anterior metasomal tergites, the shape of the first of which resembles *Cotesia*, the second *Glyptapanteles*.

*Protapanteles* is in many respects intermediate morphologically between *Cotesia* and *Glyptapanteles*. It shares a quadrate first metasomal tergite (Figure 12B) with *Cotesia*, and a weakly sculptured propodeum (Figure 12A) and more triangular second metasomal tergite (Figure 12B) with *Glyptapanteles*. An open forewing areolet and short ovipositor are shared by all three genera.

### *Sathon* Mason (Figure 13)

This relatively small genus was placed by Mason (1981) in his tribe Microgastrini, thus implying a close relationship with *Hypomicrogaster* among the genera mentioned above. However, subsequent molecular phylogenetic studies have shown that *Sathon* falls among the lineages related to *Glyptapanteles* (Whitfield et al. 2002), despite its much longer ovipositor. It will likely be subsumed within the latter genus in future. In the Ecuador project, *Sathon* has been recovered seven times, from Pyralidae on *Urtica* sp. (Urticaceae). A taxonomic revision of the genus (Williams 1988), completed before most of these neotropical rearing surveys were conducted, is likely in need of an update.

**Figure 13.**
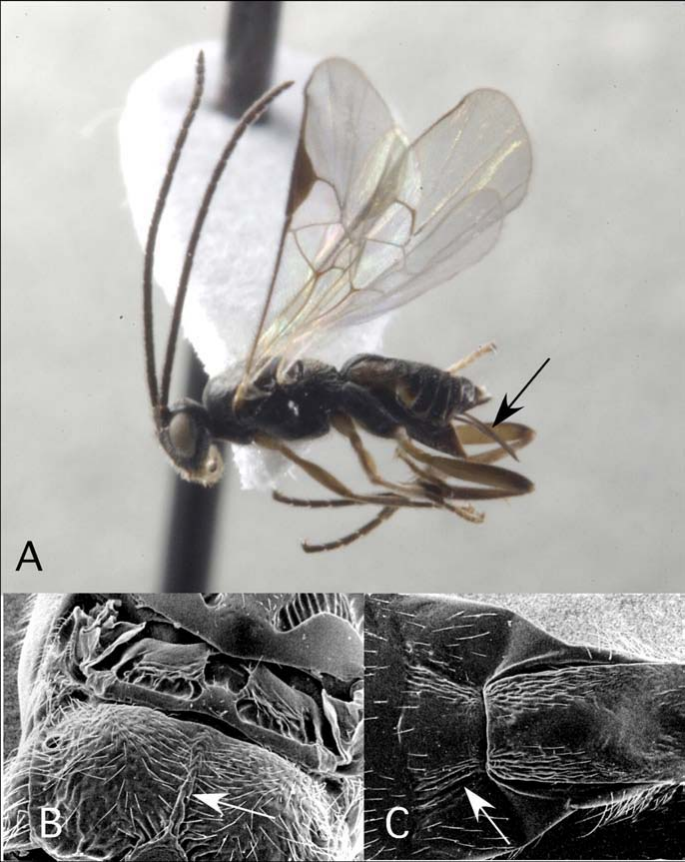
*Sothon* Mason. A: lateral habitus of a female from Yanayacu, showing relatively long ovipositor and sheaths; the hypopygium (subgenital plate) is fully sclerotized medially, unlike most other genera that have long ovipositors. B-C: diagnostic features of the genus. B: propodeum, showing medial carina. C: anterior metasomal tergites, resembling many *Glyptapanteles* in form.

*Sathon* generally resembles *Glyptapanteles*, sharing the open forewing areolet, the finely sculptured propodeum, usually with a medial carina (Figure 13B), and the posteriorly narrowing first metasomal tergite and posteriorly broadening second metasomal tergite (Figure 13C). It differs in having a long ovipositor (Figure 13A). Molecular and morphological phylogenetic studies (Whitfield et al. 2002) suggest that *Sathon* may be a subclade within *Glyptapanteles*, implying that it should ultimately become a synonym of the latter.

### *Venanus* Mason (Figure 14)

This relatively small genus is mostly known from the Andes, with rare records of it extending as far north as Canada. Mason (1981) recognized five species of *Venanus*, but additional species are currently being described by the first author and Claus Rasmussen. Confirmed host records so far are from Pyralidae or microlepidoptera on a wide variety of plants. *Venanus* has been recovered eight times from the Ecuador project from Pyralidae on Urticaceae, Boraginaceae and Cecropiaceae. One record from an arctiid on *Verbesina* (Asteraceae) needs confirmation.

**Figure 14.**
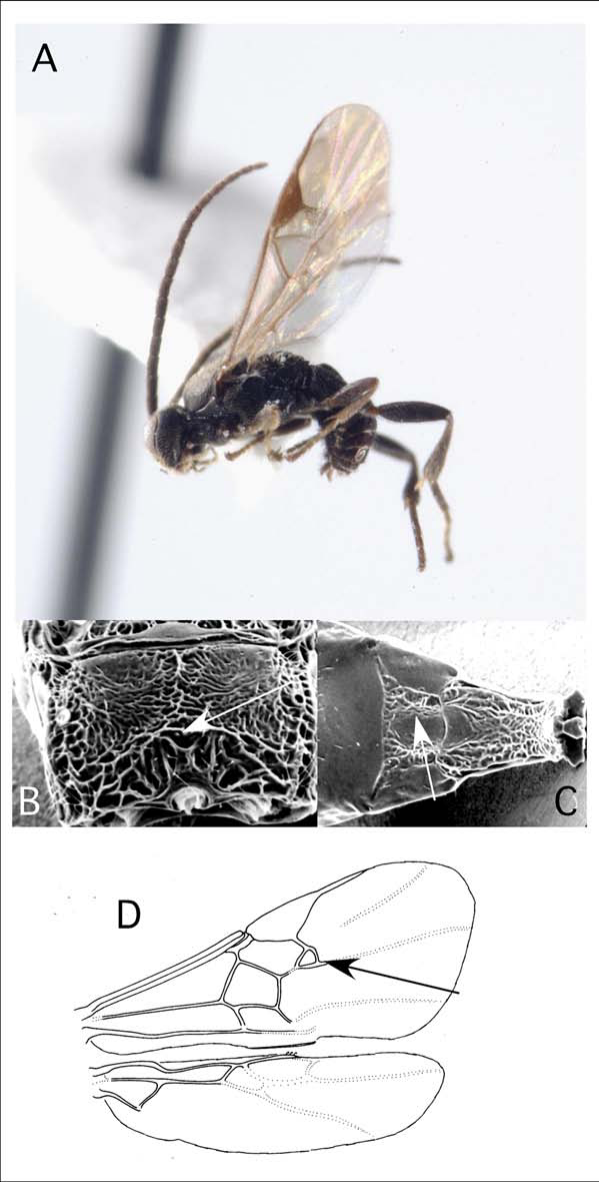
*Venanus* Mason. A: lateral habitus of a male from Yanayacu (females appear similar since they have a short ovipositor). B-D: diagnostic features of the genus. B: propodeum, showing roughly indicated transverse carina bisected by a medial carina. C: anterior metasomal tergites, showing relatively broad tergite I and relatively narrower tergite II. D: wing venation, showing distally closed small areolet in forewing (after Whitfield 1997a).

The tiny size of *Venanus* species is relatively distinctive among microgastrines. The genus can be distinguished by the following combination of features: fore wing with second r-m vein present, so that a small to moderate sized areolet is formed (Figure 14D); propodeum with medial carina rather than medial areola, usually crossing a vaguely defined transverse carina in the posterior half (Figure 14B); hind coxa relatively small, barely if at all extending beyond end of metasomal tergite I; metasomal tergite I parallel-sided to broadening posteriorly, and tergite II much narrower and more or less trapezoidal (Figure 14C); ovipositor and sheaths short and barely exserted.

### Microgastrine genera not yet recovered, but likely to occur in Ecuador

Several genera will almost certainly be reared from future sampling: *Alphomelon* Mason (from Hesperiidae Deans et al. 2003); *Deuterixys* Mason (from *Bucculatrix* and related microleps - Whitfield and Oltra Moscardo 2004); *Microplitis* Förster (from Noctuidae and Sphingidae - Janzen et al. 2003); *Promicrogaster* Brues and Richardson (from hosts feeding on bracket fungi), *Pseudapanteles* Ashmead (from gelechiid leafminers on Solanaceae, and from Crambidae), *Rasivalva* Mason (primarily from exposed macrolepidoptera, including Limacodidae) and *Rhygoplitis* Mason. This last genus is fairly common in Malaise trap samples from eastern Ecuador, and Costa Rica, but for some reason never has appeared in either of the rearing surveys. The hosts appear to be concealed microlepidoptera, including crambids.

Additional microgastrine genera that could potentially be recovered, but are more common in lowland sites, include: *Clarkinella* Mason, *Dasylagon* Muesebeck, *Fornicia* Brulle, *Larissimus* Nixon, *Prasmodon* Nixon, *Sendaphne* Nixon, *Snellenius* Westwood, *Venanides* Mason, *Wllkinsonellus* Mason and *Xanthomicrogaster* Cameron. Some of these genera are associated with either Crambidae or Limacodidae. The latter family is not well represented at Yanayacu, but becomes more diverse at lower elevations nearby.

### Hyperparasitoids of Microgastrinae recovered at Yanayacu *Mesochorus* Gravenhorst (Figure 15)

*Mesochorus*, a large genus in the family Ichneumonidae, is composed of hyperparasitoids whose hosts include a wide range of microgastrine and other ichneumonoid genera (Dasch 1974). Several *Mesochorus* have been reared from Microgastrinae hosts, including members of *Glyptapanteles* and *Hypomicrogaster*, thus far in the Yanayacu project.. In addition to microgastrine braconids, *Mesochorus* have been reared from Rogadinae. The host list for *Mesochorus* from the ACG survey in Costa Rica includes not only *Glyptapanteles* and *Hypomicrogaster*, but also *Apanteles, Diolcogaster, Cotesia, Microplitis, Parapanteles* and *Alphomelon*. The Costa Rican sampling has been more intensive and has taken place over a longer time period, but the Yanayacu *Mesochorus* samples nevertheless seem less diverse. This could perhaps be due to the lower population densities of their microgastrine hosts at the Ecuadorian site.

**Figure 15.**
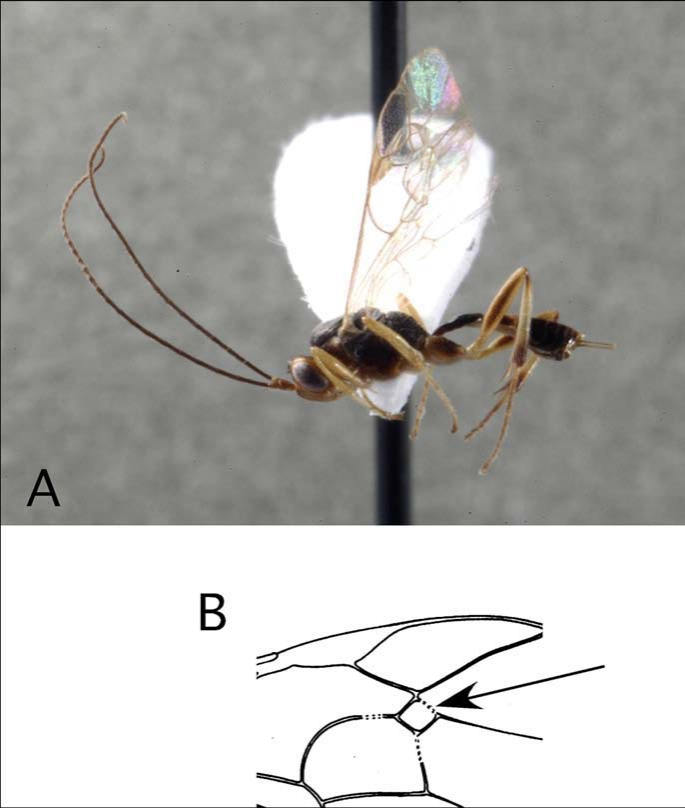
*Mesochorus* Gravenhorst. A: Lateral habitus of a male from Yanayacu. B: detail of forewing, showing characteristic diamond-shaped areolet (after Wahl 1993).

It is beyond the scope of this paper to provide means for separating *Mesochorus* from all other ichneumonid wasps, but the diamond-shaped forewing areolet (Figure 15B) is diagnostic.

### Perilampidae *(Perilampus* sp.)

One record of a perilampid wasp (*Perilampus* sp.) from microgastrine cocoons has been recorded so far, but the identity of the host genus is uncertain.

## Editor's note

Paper copies of this article will be deposited in the following libraries. Senckenberg Library, Frankfurt Germany; National Museum of Natural History, Paris, France; Field Museum of Natural History, Chicago, Illinois USA; the University of Wisconsin, Madison, USA; the University of Arizona, Tucson, Arizona USA; Smithsonian Institution Libraries, Washington D.C. USA; The Linnean Society, London, England.
